# Editorial Comment to Small renal cell carcinoma accompanied by extensive inferior vena cava tumor thrombus diagnosed by percutaneous transvenous biopsy

**DOI:** 10.1002/iju5.12676

**Published:** 2023-12-12

**Authors:** Tsunenori Kondo

**Affiliations:** ^1^ Department of Urology Tokyo Women's Medical University Adachi Medical Center Tokyo Japan

Renal cell carcinoma (RCC) sometimes extends to the inferior vena cava (IVC), with an incidence of up to 10%.^1^ However, other diseases, such as leiomyosarcoma or urothelial carcinoma, may also form a tumor thrombus in the IVC.^2^ Surgical resection is a mainstay of treatment for patients with tumor thrombus, irrespective of the tumor histology. Some tumors are too complicated to be resected because of the invasion to surrounding tissues or wide tumor spread. In such cases, a definite diagnosis is necessary to consider systemic therapy. Biopsy to the tumors is not so difficult when a primary tumor is large enough and easy to access through percutaneous biopsy. However, biopsy is difficult if the primary tumors are too small to sample tissues.

In this study, the authors reported a case of small RCC extending into the IVC and the right atrium.^3^ Initially, the authors speculated that the tumor thrombus may be leiomyosarcoma since the renal tumor was just 1.5 cm in size. The pathological diagnosis was made with a percutaneous biopsy to the renal vein. I think that the authors have reported an interesting case.

Biopsy to tumors in IVC or renal veins can be done through a few approaches. The authors chose percutaneous ultrasound‐guided transvenous biopsy. This technique can be done in any institutes. However, there may be some downsides to this method, including difficulty of accurate targeting, because of the long distance from the body surface and respiratory movement. Computed tomography‐guided biopsy may improve the accuracy of targeting, and this method is currently often selected. On the other hand, major hemorrhage has been reported as one of the serious complications of percutaneous biopsy of solid organs, with a reported incidence of 0.1–8.3%.^4^ Another possible option is endovascular biopsy, which has been reported as an emerging technique alternative to the percutaneous approach.^5^ This technique can be done with local anesthesia, and is associated with lower risk of massive hemorrhage, according to a previous report.^5^ Growing evidence will allow for the evaluation of its feasibility soon.

Nevertheless, I believe that this case report reminds us of the importance of making a pathological diagnosis when performing systemic therapy in the era of histology‐tailored treatment. Currently, biopsy for tumors in the IVC or renal veins has become more accessible. It is our responsibility to be aware of the various approaches to these tumors.

## Author contributions

Tsunenori Kondo: Conceptualization; writing – original draft; writing – review and editing.

## Conflict of interest

Tsunenori Kondo received honoraria from Pfizer, MSD, Takeda Pharmaceutical Company, and Ono Pharmaceutical.
